# Efficacies and Toxicities of Seven Chemotherapy Regimens for Advanced Hodgkin Lymphoma

**DOI:** 10.3389/fphar.2021.694545

**Published:** 2021-11-16

**Authors:** Fajun Pei, Yang Yu, Bin Dong, Hui Guan, Xinzhe Dong, Fen Zhao

**Affiliations:** ^1^ Department of Urology Surgery, Shandong Cancer Hospital and Institute, Shandong First Medical University and Shandong Academy of Medical Sciences, Jinan, China; ^2^ School of Graduate Studies, Shandong Academy of Medical Sciences, Shandong First Medical University, Jinan, China; ^3^ Department of Radiation Oncology, Shandong Cancer Hospital and Institute, Shandong First Medical University and Shandong Academy of Medical Sciences, Jinan, China; ^4^ Department of Medical Imaging, The First People’s Hospital of Pingdu, Qingdao, China; ^5^ Department of Radiation Oncology, The Fourth People’s Hospital of Jinan, Jinan, China; ^6^ Department of Radiation Oncology, Qilu Hospital of Shandong University, Jinan, China; ^7^ Shandong University, Jinan, China

**Keywords:** hodgkin lymphoma, chemotherapy, efficacy, randomized controlled trial, network meta-analysis

## Abstract

**Background/Aims:** Hodgkin Lymphoma (HL) has become one of the most treatable cancers, with more than 80% patients in the advanced stage being cured through improvement of therapeutic regimens. Nevertheless, some treatments were accompanied with toxicities.

**Methods:** In the current study, a network meta-analysis (NMA) was conducted to compare the efficacies and toxicities of different chemotherapy regimens for advanced Hodgkin lymphoma (HL). We reviewed PubMed and EMBASE databases from inception to May 2018, and identified randomized controlled trials (RCTs) in which advanced HL patients received chemotherapy. Fourteen eligible RCTs published between 1992 and 2017 were enrolled in this NMA. These studies included a total of 5,964 HL patients, and assessed at least one of seven different chemotherapy regimens. Direct and indirect evidence was combined to calculate odds ratios (ORs) and 95% confidence intervals (95% CIs), and to establish a surface under the cumulative ranking (SUCRA) curve.

**Results:** A cluster analysis was performed to evaluate efficacies and toxicities of different regimens. The COPP + ABVD (cyclophosphamide + vincristine + procarbazine + prednisone + doxorubicin + bleomycin + vinblastine + dacarbazine) regimen had the highest SUCRA partial response and overall remission rate values, while the ABVD regimen resulted in the lowest incidences of anemia, thrombocytopenia, neutropenia, and leucopenia.

**Conclusion:** Cluster analysis revealed that COPP + ABVD had the best efficacy against advanced HL among the seven regimens, and ABVD had the lowest toxicity.

## Introduction

Hodgkin lymphoma (HL) is a malignancy of the lymphatic system with characteristics that the presence of Reed-Sternberg cells, although these cells typically account for <1% of cells in the affected tissue (Yung and Linch, 2003). The disease is more common in men than women, and peaks in incidence in young adults and in those whose age was older than 60 years (Townsend and Linch, 2012). HL is rare among children and relatively rare in middle-aged adult, but is the most commonly diagnosed cancer among adolescents in the age range 15–19 (Punnett et al., 2010). Five-year survival for HL patients during the 2000–2004 periods was 85.2% (Shenoy et al., 2011).

Advanced stage HL is usually treated with chemotherapy and radiotherapy (Eichenauer et al., 2011). The MOPP (mechlorethamine + vincristine + procarbazine + prednisone) chemotherapy regimen was developed to treat patients with advanced HL following radiation (Ansell, 2016; Vassilakopoulos and Johnson, 2016), and results in a long-term progression-free survival (PFS) rate of 54% and an overall survival (OS) rate of 48% (Ansell, 2016). The combination of doxorubicin, bleomycin, vinblastine and dacarbazine (ABVD) is currently the standard of treatment for HL around the world (Chisesi et al., 2011; Corazzelli et al., 2011). ABVD was considered as a better option for HL therapy when compared to non-cross-resistant alternating regimens (MOPP + ABV [hybrid]: mechlorethamine + vincristine + procarbazine + prednisone + doxorubicin + bleomycin + vinblastine, as it was associated with a higher risk of secondary malignancy (Souza et al., 2009). However, recent findings suggest that the BEACOPP (bleomycin + etoposide + doxorubicin + cyclophosphamide + vincristine + procarbazine + prednisone) regimen is more effective in controlling advanced HL than ABVD (Corazzelli et al., 2011). The dose-dense Stanford V (doxorubicin + vinblastine + mechlorethamine + vincristine + bleomycin + etoposide + prednisone) regimen was developed in the early 1990s, but ultimately failed to improve upon ABVD outcomes (Chisesi et al., 2011; Vassilakopoulos and Johnson, 2016). The German Hodgkin Study Group (GHSG) developed the COPP + ABVD regimen (cyclophosphamide + vincristine + procarbazine + prednisone + doxorubicin + bleomycin + vinblastine + dacarbazine) to improve advanced HL patient outcomes (Vassilakopoulos and Johnson, 2016).

While many chemotherapy regimens are used for treatment of advanced HL, the optimal regimen has not yet been identified. We conducted a systematic review including bothtraditional and network meta-analyses (NMA) to more precisely evaluate the efficacies and toxicities of various HL chemotherapy regimens. And these regimens we choosed which are commonly used in developing countries now and so this results would be of most use to them.

## Materials and Methods

### Study Retrieval Strategy

The PubMed and EMBASE electronic databases were comprehensively searched from inception to May 2018. The search strategy was combined key words and free words, including the following search terms: “lymphoma,” “chemotherapy regimen,” “doxorubicin,” “bleomycin,” “vinblastine,” “dacarbazine,” “etoposide,” “cyclophosphamide,” “vincristine,” “procarbazine,” “prednisone,” “mechlorethamine,” and “randomized controlled trial” (RCT). We also conducted a manual search to identify additional relevant references (Supplementary Material).

### Inclusion and Exclusion Criteria

Studies inclusion criteria were as follows: 1) RCTs; 2) different interventions for treating HL were included in this study such as ABVD: combination of doxorubicin, bleomycin, vinblastine and dacarbazine; BEACOPP: an intensified regimen consisting of bleomycin, etoposide, doxorubicin, cyclophosphamide, vincristine, procarbazine, and prednisone; Stanford V: doxorubicin, vinblastine, mechlorethamine, vincristine, bleomycin, etoposide and prednisone; COPP + ABVD: cyclophosphamide, vincristine, procarbazine, prednisone, doxorubicin, bleomycin, vinblastine and dacarbazine; MOPP: mechlorethamine, vincristine, procarbazine and prednisone; MOPP + ABV (Hybrid): mechlorethamine, vincristine, procarbazine, prednisone, doxorubicin, bleomycin and vinblastine; MOPP + ABVD (Alternating): mechlorethamine, vincristine, procarbazine and prednisone followed by doxorubicin, bleomycin, vinblastine and dacarbazine; 3) advanced HL patients (untreated advanced HL) were diagnosed by histopathological examination, and aged 16–83 years; 4) outcomes that including CR, PR, ORR, OS, anemia, thrombocytopenia, neutropenia, leukopenia and nausea/vomiting were described; and 5) study was published in English. Exclusion criteria were: 1) patients with severe heart or lung diseases and metabolic diseases; 2) pregnant or lactating patients; 3) patients who received prior radiotherapy or chemotherapy; 4) nodular lymphocyte predominant HL; 5) patients with hepatic or renal dysfunction; 6) studies with insufficient data, such as non-paired studies; 7) non-RCT studies; 8) duplicated publications; 9) meeting reports, systematic reviews (meta-analyses), or summaries and (10) non-English literature, 11) Studies containing targeted protocols.

### Data Extraction and Quality Assessment

Data were collected from enrolled studies by two researchers independently using a unified data collection form. Any disagreements in the data extraction process were resolved by discussion. Two researchers evaluated method quality for each RCT according to the Cochrane Collaboration’s tool for assessing bias risk (Higgins et al., 2011). This tool comprises random method, allocation concealment, blinding, attrition, selective reporting and other bias. The assessment designates a value of ‘‘low,’’ ‘‘high,’’ or ‘‘unclear’’ risk of bias by assigning a judgment of “yes,” “no,” or “unclear,” respectively, for each domain. The number of domains deemed “unclear” or “no” is calculated and each study is classified as follows: 1) 0–1 domains, low bias risk; 2) ≥4 domains, high bias risk; 3) 2–3 domains, moderate bias risk (Chung and Lee, 2013). Quality assessments and investigation of publication bias were performed using Review Manager 5 (RevMan 5.2.3, Cochrane Collaboration, Oxford, United Kingdom).

### Statistical Analysis

Direct comparisons between different treatment arms were made using a traditional pairwise meta-analysis. Odds ratios (ORs) and 95% confidence intervals (CIs) were used to combine intervention efficacy estimates. Study heterogeneity was examined using Chi-square and I-square tests (Chen et al., 2015). Results were presented as a network plot using R version 3.2.2, with each node indicating an intervention, node sizes representing sample sizes, and the thickness of lines connecting any two nodes representing the number of included studies. Comparisons between different interventions were made using Bayesian NMA. According to non-informative priors, effect sizes and precision were specified in each analysis. After four chains and a 20,000-simulation burn-in phase, convergence and lack of auto-correlation were explored and verified. Direct probability statements were concluded in an additional 50,000-simulation phase (Tu et al., 2012). The node-splitting method was used to select a consistency or inconsistency model, by evaluating the consistency between direct and indirect evidence (Zhu et al., 2015). For the interpretation of ORs, the probability of each intervention being the most effective or safest treatment was calculated by using a Bayesian approach, with probability values estimated by the surface under the cumulative ranking (SUCRA) curve and the rank of each intervention (Salanti et al., 2011; Chaimani et al., 2013). Cluster analysis was used to group the short-term efficacies and toxicities of regimens according to their similarity (Chaimani et al., 2013). R v3.2.1 package gemtc (V.0.6) with the Markov Chain Monte Carlo engine Open BUGS (V.3.4.0) were used for all calculations in this study.

## Results

### Baseline Characteristics of Included Studies

**FIGURE 1:**
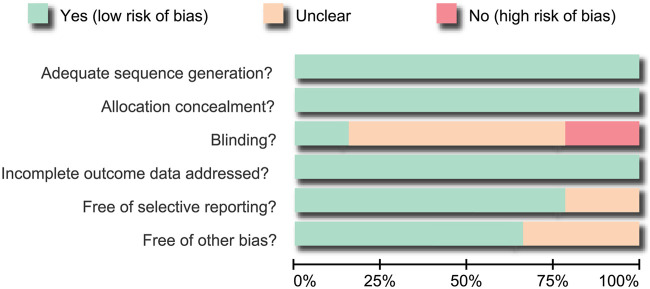
Bias risk assessment using the Cochrane Collaboration’s tool. Fourteen eligible randomized controlled trials were analyzed in this NMA.

A total of 1,088 articles studying HL cases treated with at least one of seven chemotherapy regimens were initially identified from electronic databases. We excluded two duplicate studies, 17 letters or reviews, 4 non-human studies, and 109 non-English articles. The remaining 965 studies were evaluated according to the full text. We further excluded 378 non-RCT studies, 377 unrelated to HL, 185 unrelated to chemotherapy, 1 with duplicated contents and 1 due to unavailable or missing data. In total, 14 RCT studies met our meta-analysis inclusion criteria. These studies evaluated seven chemotherapy regimens, including ABVD, BEACOPP, Stanford V, MOPP, COPP + ABVD, MOPP + ABV (hybrid), and MOPP + ABVD (alternating) (Canellos et al., 1992; Somers et al., 1994; Connors et al., 1997; Glick et al., 1998; Diehl et al., 2003; Duggan et al., 2003; Ballova et al., 2005; Gobbi et al., 2005; Federico et al., 2009; Hoskin et al., 2009; Viviani et al., 2011; Gordon et al., 2013; Mounier et al., 2014; Carde et al., 2016) (Supplementary Figure S1). They included 5,964 total Caucasian patients with HL, most of whom received the ABVD regimen. Included RCTs were published between 1992 and 2016. The enrolled studies included 14 study objects from European and American, 13 two-arm trials and 1 was a three-arm trail. Characteristics of included studies are shown in Supplementary Table S1 and the bias assessment with Cochrane Collaboration’s tool is presented in Figure 1. In the assessment of blinding, some studies were considered has having an unclear bias for missing the discussion over the blinding-related results, some had a high risk of bias for the incompleteness of blinding, and there was an unclear risk of other bias as they did not describe patients lost to follow-up. In selective reporting, some studies were characterized as unclear risk as they did not give the explicit description about whether pre-specified outcomes had been shown.

### Pairwise Meta-Analysis to Assess Efficacies and Toxicities of the Seven Chemotherapy Regimens

Direct pairwise comparisons of efficacies and toxicities of the seven chemotherapy regimens have carried out. As shown in Table 1, in terms of the short-term efficacy, patients treated with the Stanford V regimen had relatively higher PR rates in the short-term (OR = 0.72, 95%CI = 0.56–0.93) than those treated with the ABVD regimen; about the long-term efficacy, MOPP had lower overall survival (HR = 1.49, 95%CI = 1.01–2.19) compared to patients treated with MOPP + ABVD. As seen in Table 2, incidences of anemia, thrombocytopenia, neutropenia, and leucopenia were higher in BEACOPP-treated patients (anemia: OR = 0.20, 95%CI = 0.09–0.43; thrombocytopenia: OR = 0.08, 95%CI = 0.03–0.21; neutropenia: OR = 0.60, 95%CI = 0.41–0.87; Leucopenia: OR = 0.13, 95%CI = 0.09–0.19) than those treated with the ABVD regimen. The MOPP (anemia: OR = 0.17, 95%CI = 0.07–0.41; thrombocytopenia: OR = 0.10, 95%CI = 0.04–0.24; neutropenia: OR = 0.31, 95%CI = 0.18–0.51) and MOPP + ABVD regimens (anemia: OR = 0.21, 95%CI = 0.08–0.51; thrombocytopenia: OR = 0.13, 95%CI = 0.05–0.31; neutropenia: OR = 0.26, 95%CI = 0.15–0.43) exhibited higher incidences of anemia, thrombocytopenia, and neutropenia, while the Stanford V regimen resulted in higher incidences of anemia and leucopenia (anemia: OR = 0.05, 95%CI = 0.01–0.42; leucopenia: OR = 0.53, 95%CI = 0.34–0.81).

**TABLE 1:** Estimated OR and 95%CI of pairwise meta-analysis for efficacy in Hodgkin lymphoma.

Included studies	Comparisons	Heterogeneity test		Pairwise meta-analysis		
		*I* ^*2*^	*P*_*h*_	OR/HR (95%CI)	*Z*	*P*
CR						
4 studies	A vs. B	0.00%	0.849	0.98 (0.83–1.16)	0.22	0.824
3 studies	A vs. C	38.0%	0.199	1.23 (0.98–1.54)	1.76	0.079
2 studies	B vs. E	0.00%	0.910	1.03 (0.83–1.28)	0.29	0.771
1 study	A vs. F	NA	NA	0.95 (0.78–1.16)	0.49	0.623
2 studies	F vs. G	0.00%	0.721	1.12 (0.92–1.37)	1.15	0.252
2 studies	D vs. G	0.00%	0.580	0.87 (0.65–1.17)	0.91	0.362
1 study	A vs. D	NA	NA	1.21 (0.82–1.79)	0.96	0.335
1 study	A vs. G	NA	NA	0.99 (0.68–1.44)	0.07	0.940
PR						
2 studies	A vs. B	0.00%	0.649	0.95 (0.47–1.90)	0.22	0.824
3 studies	**A vs. C**	**3.80%**	**0.354**	**0.72(0.56–0.93)**	**2.48**	**0.013**
1 study	B vs. E	NA	NA	0.09 (0.00–1.79)	0.29	0.771
1 study	A vs. F	NA	NA	1.31 (0.87–1.97)	0.49	0.623
2 studies	F vs. G	33.80%	0.219	0.87 (0.64–1.18)	1.15	0.252
2 studies	D vs. G	85.80%	0.008	1.20 (0.76–1.89)	0.91	0.362
1 study	A vs. D	NA	NA	0.55 (0.28–1.08)	0.96	0.335
1 study	A vs. G	NA	NA	1.34 (0.60–2.98)	0.07	0.940
ORR						
2 studies	A vs. B	0.00%	0.934	0.94 (0.72–1.23)	0.43	0.666
3 studies	A vs. C	0.00%	0.905	1.03 (0.88–1.19)	0.36	0.722
1 study	B vs. E	NA	NA	0.86 (0.42–1.78)	0.40	0.687
1 study	A vs. F	NA	NA	0.99 (0.82–1.21)	0.07	0.944
2 studies	F vs. G	0.00%	0.999	1.06 (0.88–1.27)	0.64	0.520
2 studies	D vs. G	0.00%	0.639	0.93 (0.71–1.22)	0.53	0.596
1 study	A vs. D	NA	NA	1.04 (0.72–1.50)	0.21	0.830
1 study	A vs. G	NA	NA	1.02 (0.71–1.47)	0.12	0.904
OS						
4 studies	A vs. B	58.60%	0.065	0.87 (0.56, 1.37)	0.59	0.553
3 studies	A vs. C	0.00%	0.477	0.87 (0.64, 1.19)	0.85	0.397
2 studies	B vs. E	35.40%	0.213	0.83 (0.60, 1.15)	1.11	0.269
1 study	A vs. F	NA	NA	0.79 (0.56, 1.10)	1.41	0.158
2 studies	F vs. G	0.00%	0.744	0.85 (0.61, 1.18)	0.96	0.337
2 studies	**D vs. G**	**0.00%**	**0.940**	**1.49 (1.01, 2.19)**	**2.00**	**0.045**
1 study	A vs. D	NA	NA	0.81 (0.48, 1.38)	0.78	0.437
1 study	A vs. G	NA	NA	1.16 (0.68, 1.97)	0.56	0.579

Notes: CR, complete response; PR, partial response; ORR, overall remission rate; HR, hazard ratio; OS, overall survival; OR, odds radio; 95%CI, 95%confidence intervals; NA, not available; A, ABVD (doxorubicin + bleomycin + vinblastine + dacarbazine); B, BEACOPP(bleomycin + etoposide + doxorubicin + cyclophosphamide + vincristine + procarbazine + prednisone); C, StanfordV(doxorubicin + vinblastine + mechlorethamine + vincristine + bleomycin + etoposide + prednisone); D, MOPP(mechlorethamine + vincristine + procarbazine + prednisone); E, COPP + ABVD(cyclophosphamide + vincristine + procarbazine + prednisone + doxorubicin + bleomycin + vinblastine + dacarbazine); F, MOPP + ABV(Hybrid) (mechlorethamine + vincristine + procarbazine + prednisone + doxorubicin + bleomycin + vinblastine); G, MOPP + ABVD(Alternating); significant difference is shown in bold and underline formats.

**TABLE 2:** Estimated OR and 95%CI of pairwise meta-analysis for toxicity in Hodgkin lymphoma.

Included studies	Comparisons	Heterogeneity test		Pairwise meta-analysis		
		*I* ^*2*^	*P*_*h*_	OR (95%CI)	*Z*	*P*
Anemia						
2 studies	A vs. B	30.4%	0.231	**0.20 (0.09–0.43)**	4.07	**0.000**
1 study	A vs. C	NA	NA	**0.05 (0.01∼0.42)**	2.79	**0.005**
2 studies	B vs. E	18.9%	0.267	**2.95 (1.75–4.98)**	4.07	**0.000**
1 study	F vs. G	NA	NA	0.89 (0.64–1.25)	0.67	0.502
1 study	A vs. D	NA	NA	**0.17 (0.07–0.41)**	3.88	**0.000**
1 study	A vs. G	NA	NA	**0.21 (0.08–0.51)**	3.39	**0.001**
1 study Thrombocytopenia	D vs. G	NA	NA	1.23 (0.72–2.10)	0.74	0.457
3 studies 1 study 2 studies	A vs. B	0.0%	0.621	**0.08(0.03–0.21)**	5.22	**0.000**
	A vs. C	NA	NA	0.88 (0.05–14.19)	0.09	0.926
	B vs. E	15.6%	0.276	**1.71 (1.01–2.90)**	1.98	**0.048**
1 study	F vs. G	NA	NA	**0.43 (0.29–0.64)**	4.18	**0.000**
1 study 1 study	A vs. D	NA	NA	**0.10 (0.04–0.24)**	5.12	**0.000**
	A vs. G	NA	NA	**0.13 (0.05–0.31)**	4.55	**0.000**
1 study	D vs. G	NA	NA	1.26 (0.81–1.97)	1.01	0.311
Neutropenia						
2 studies	A vs. B	0.0%	0.722	**0.60 (0.41–0.87)**	2.70	**0.007**
1 study	A vs. C	NA	NA	0.85 (0.48–1.49)	0.57	0.570
1 study	F vs. G	NA	NA	1.09 (0.86–1.38)	0.68	0.494
1 study	A vs. D	NA	NA	**0.31 (0.18–0.51)**	4.47	**0.000**
1 study	A vs. G	NA	NA	**0.26 (0.15–0.43)**	5.20	**0.000**
1 study	D vs. G	NA	NA	0.84 (0.57–1.23)	0.89	0.372
Leucopenia						
3 studies	A vs. B	89.8%	0.000	**0.13(0.09–0.19)**	11.23	**0.000**
2 studies	A vs. C	40.4%	0.254	**0.53 (0.34–0.81)**	2.94	**0.003**
2 studies	B vs. E	0.0%	0.859	1.03 (0.82–1.29)	0.27	0.786
Nausea/vomiting						
1 study	A vs. C	NA	NA	1.32 (0.36–4.79)	0.42	0.677
1 study	A vs. B	NA	NA	1.61 (0.64–4.05)	1.01	0.313
1 study	B vs. E	NA	NA	**0.60 (0.40–0.90)**	2.49	**0.013**
1 study	F vs. G	NA	NA	**0.53 (0.33–0.87)**	2.55	**0.011**
1 study	A vs. D	NA	NA	1.20 (0.71–2.03)	0.66	0.508
1 study	A vs. G	NA	NA	0.85 (0.52–1.39)	0.66	0.511
1 study	D vs. G	NA	NA	0.71 (0.43–1.17)	1.34	0.181

Notes: OR, odds radio;95%CI, 95%confidence intervals; NA, not available; A, ABVD (doxorubicin + bleomycin + vinblastine + dacarbazine), B, BEACOPP (bleomycin + etoposide + doxorubicin + cyclophosphamide + vincristine + procarbazine + prednisone), C, StanfordV (doxorubicin + vinblastine + mechlorethamine + vincristine + bleomycin + etoposide + prednisone), D, MOPP(mechlorethamine + vincristine + procarbazine + prednisone), E, COPP + ABVD (cyclophosphamide + vincristine + procarbazine + prednisone + doxorubicin + bleomycin + vinblastine + dacarbazine), F, MOPP + ABV(Hybrid) (mechlorethamine + vincristine + procarbazine + prednisone + doxorubicin + bleomycin + vinblastine), G, MOPP + ABVD(Alternating); significant difference is shown in bold and underline formats.

### Network Evidence of Seven Chemotherapy Regimens in the Treatment of HL

**FIGURE 2:**
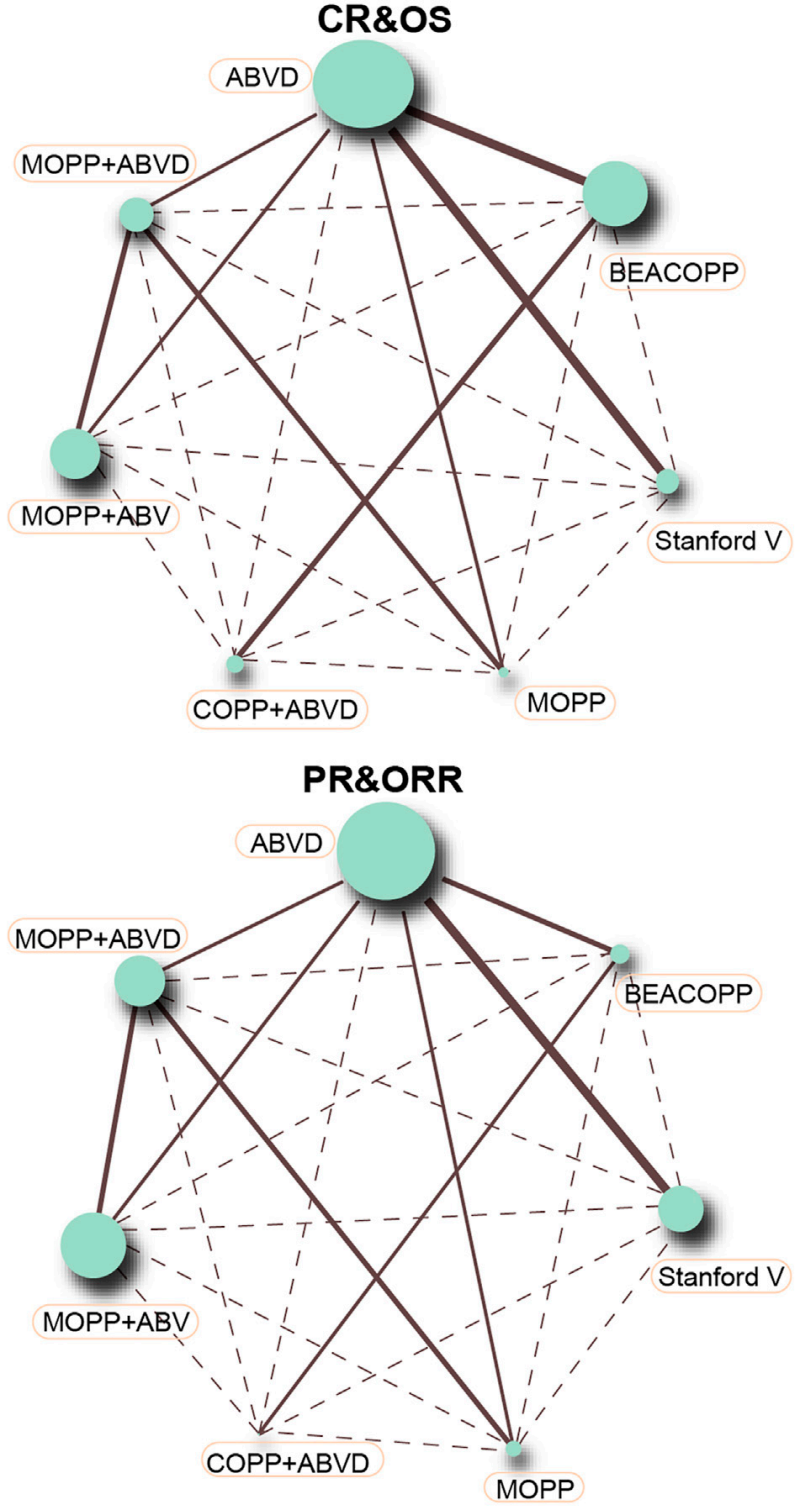
CR, PR, ORR, and OS in advanced HL patients treated with different chemotherapy regimens (each node represented an intervention, node sizes implicated sample sizes, and the thickness of lines connecting any two nodes signified the number of included studies).

**FIGURE 3:**
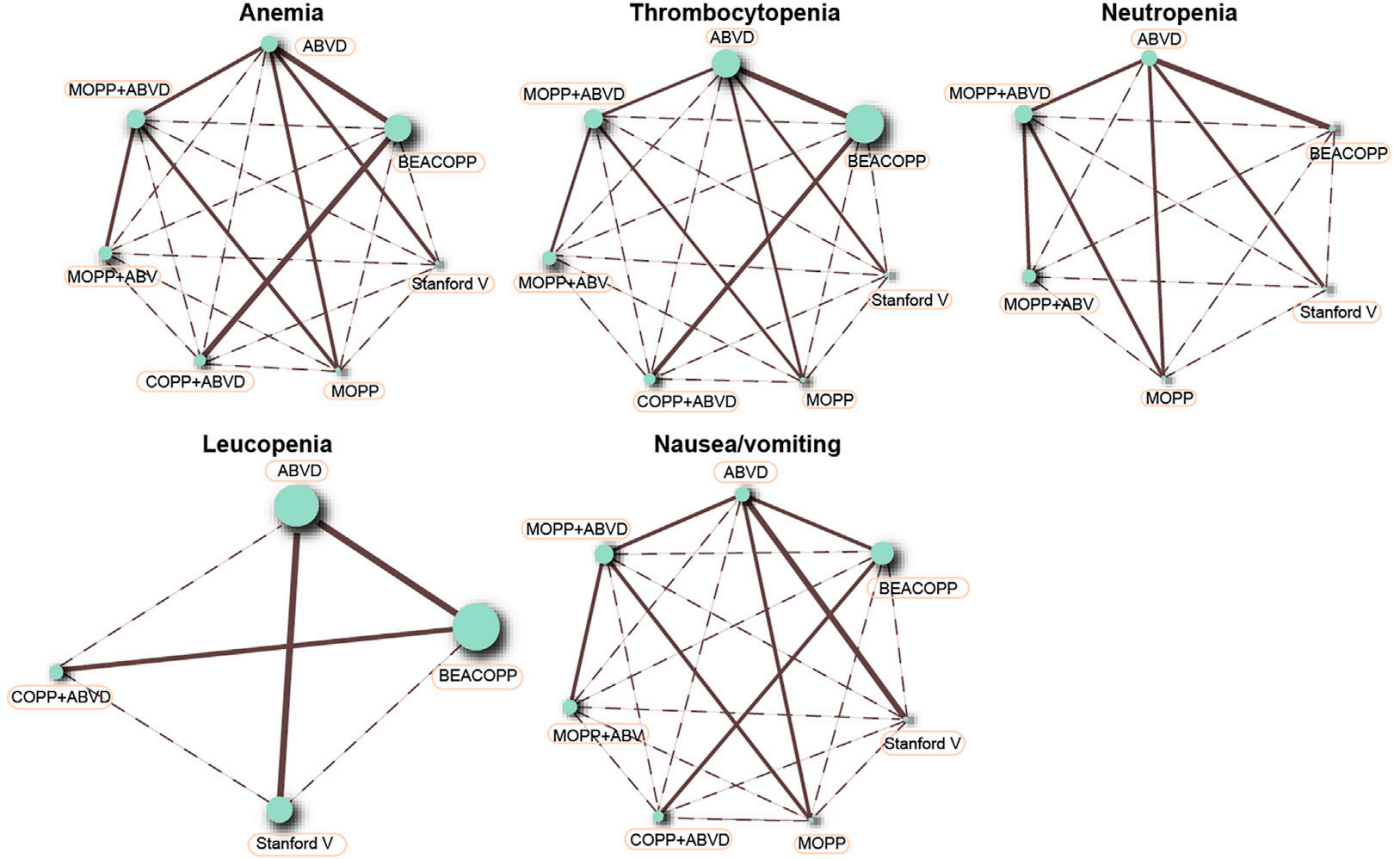
Anemia, thrombocytopenia, neutropenia, leucopenia, and nausea/vomiting in advanced HL patients treated with different chemotherapy regimens.

This NMA included seven chemotherapy regimens, including, ABVD, BEACOPP, Stanford V, COPP + ABVD, MOPP, MOPP + ABV (Hybrid) and MOPP + ABVD (Alternating). It could be observed in Figure 2 that at the aspect of chemotherapy efficacy, as CR and OS, the majority of HL patients received the ABVD regimen followed by MOPP + ABVD regimen and there were more studies focusing on ABVD *vs* BEACOPP and ABVD *vs* Stanford V. As shown in Figure 3, at the aspect of toxicity, as anemia, thrombocytopenia, leucopenia and nausea/vomiting, BEACOPP regimen was selected by most of the HL patients; for neutropenia, MOPP + ABVD regimen was selected by the great majority of HL patients and there were more studies focusing on ABVD *vs* BEACOPP.

### Inconsistency Tests of CR, PR, ORR, and OS Among all Included Studies

**FIGURE 4:**
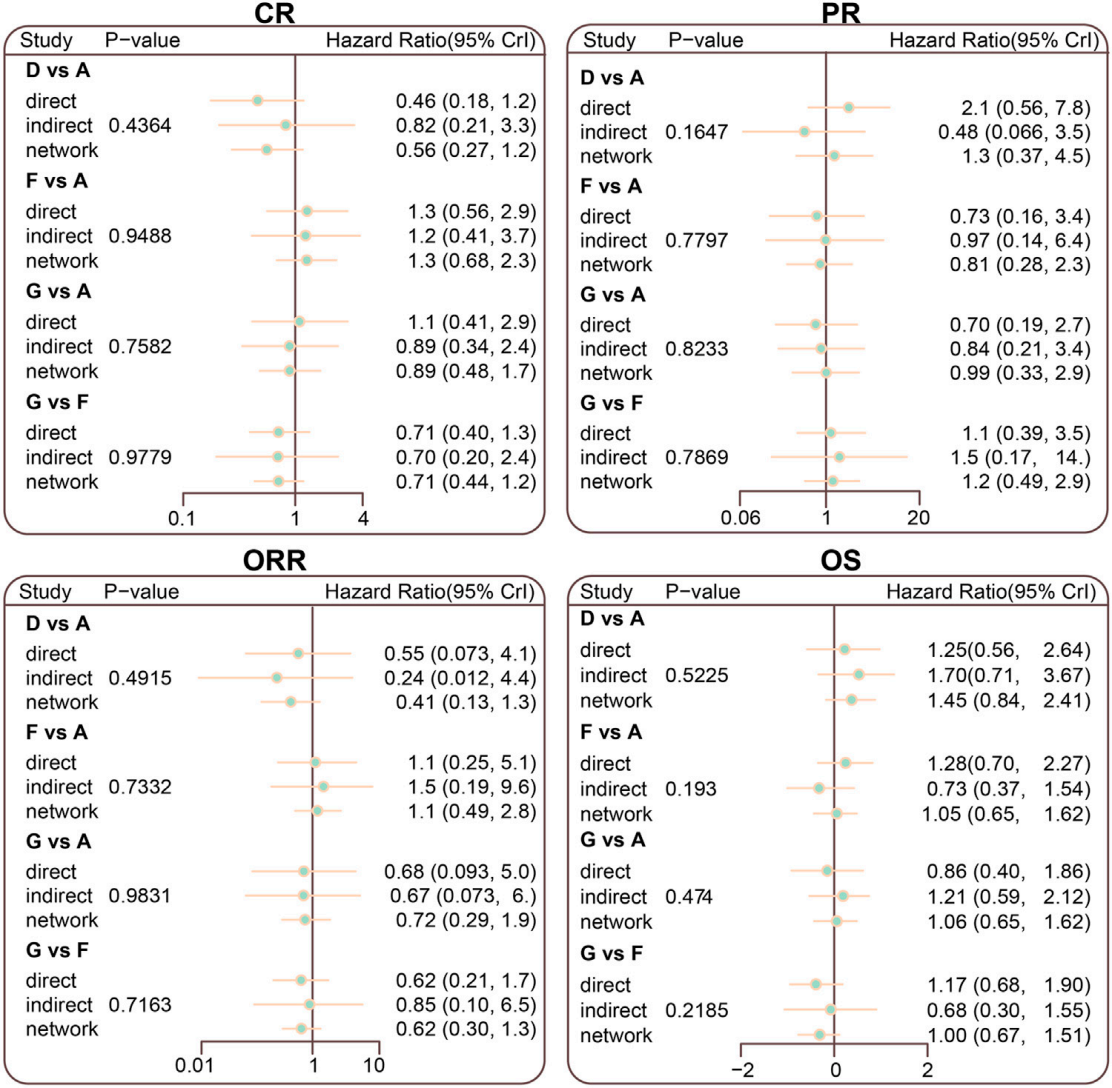
Node splitting diagram for CR, PR, ORR, and OS in advanced HL patients treated with different chemotherapy regimens; A = ABVD (doxorubicin + bleomycin + vinblastine + dacarbazine); B = BEACOPP (bleomycin + etoposide + doxorubicin + cyclophosphamide + vincristine + procarbazine + prednisone); C = StanfordV (doxorubicin + vinblastine + mechlorethamine + vincristine + bleomycin + etoposide + prednisone); D = MOPP (mechlorethamine + vincristine + procarbazine + prednisone);E = COPP + ABVD (cyclophosphamide + vincristine + procarbazine + prednisone + doxorubicin + bleomycin + vinblastine + dacarbazine); F = MOPP + ABV (Hybrid) (mechlorethamine + vincristine + procarbazine + prednisone + doxorubicin + bleomycin + vinblastine); G = MOPP + ABVD (Alternating).

The inconsistency tests of CR, PR, ORR, and OS were performed using the node-splitting method. Consistency was shown in direct and indirect evidence of all outcomes, and thus the consistency model was adopted (all *p* > 0.05) (Figure 4).

### Main Results of Network Meta-analyses

**FIGURE 5:**
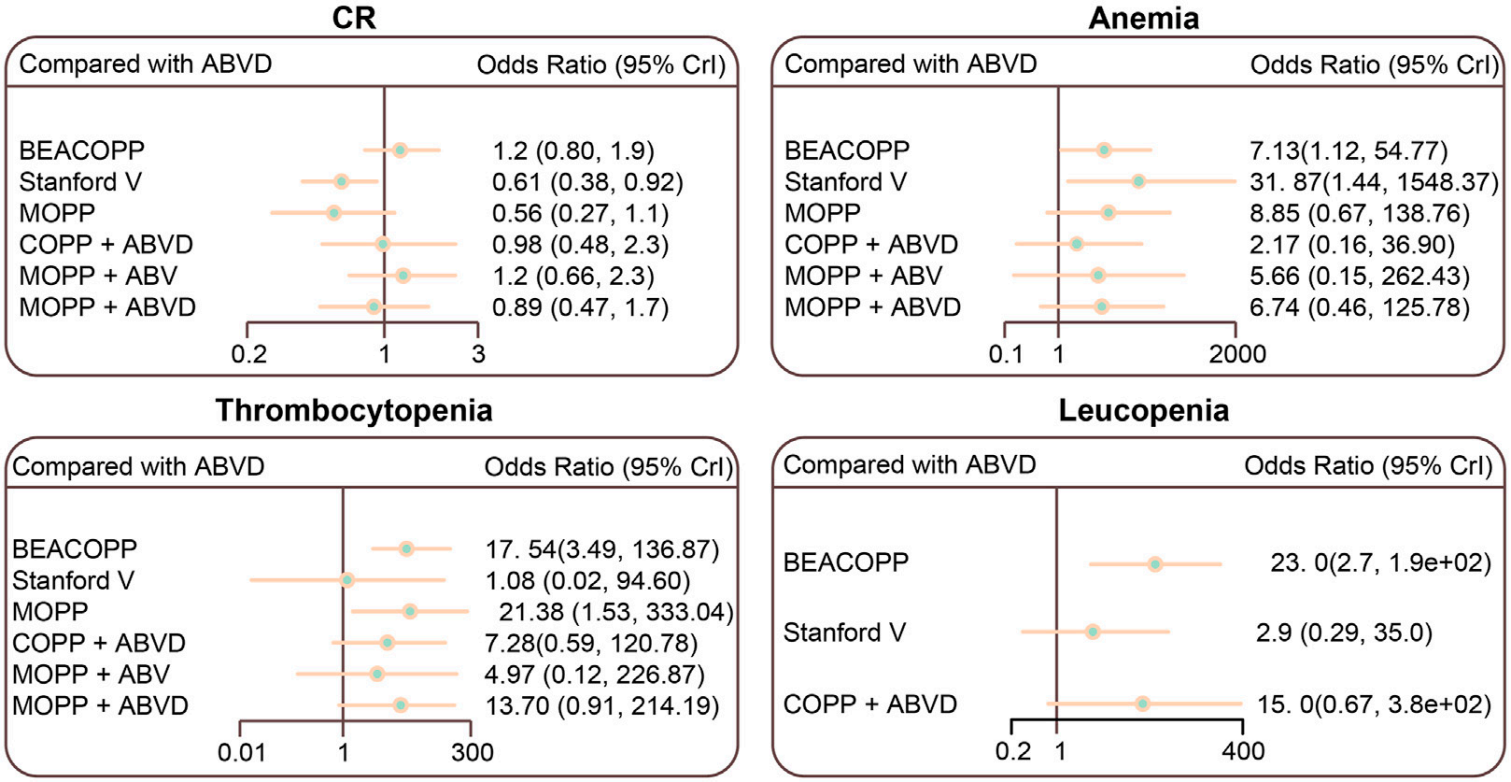
Relative relationship forest plots for CR, anemia, thrombocytopenia, and leucopenia in advanced HL patients treated with different chemotherapy regimens. ABVD (doxorubicin + bleomycin + vinblastine + dacarbazine); BEACOPP (bleomycin + etoposide + doxorubicin + cyclophosphamide + vincristine + procarbazine + prednisone); Stanford V (doxorubicin + vinblastine + mechlorethamine + vincristine + bleomycin + etoposide + prednisone); MOPP (mechlorethamine + vincristine + procarbazine + prednisone); COPP (cyclophosphamide + vincristine + procarbazine + prednisone); ABV (Hybrid) (doxorubicin + bleomycin + vinblastine).

The Stanford V regimen resulted in lower CR rates than the ABVD regimen (OR = 0.61, 95% CI = 0.38–0.92). The BEACOPP and Stanford V regimens had higher anemia incidences than the ABVD regimen (OR = 7.13, 95% C I = 1.12–54.77; OR = 31.87, 95% CI = 1.44–1548.37, respectively). Thrombocytopenia occurred more frequently in BEACOPP- and MOPP-treated HL patients than those treated with the ABVD regimen (OR = 17.54, 95% CI = 3.49–136.87; OR = 21.38, 95% CI = 1.53–333.04, respectively). Leucopenia occurred more frequently in BEACOPP-treated patients than ABVD-treated patients (OR = 23.00, 95% CI = 2.70–1.9e+02) (Figure 5; Table 3; Supplementary Table S2).

**TABLE 3:** OR (95%CI) of seven treatment modalities of three endpoints for efficacy in Hodgkin lymphoma.

OR/HR (95%CI)						
CR						
ABVD	1.2 (0.80, 1.9)	**0.61 (0.38, 0.92)**	0.56 (0.27, 1.1)	0.98 (0.48, 2.3)	1.2 (0.66, 2.3)	0.89 (0.47, 1.7)
0.83 (0.53, 1.3)	BEACOPP	**0.50 (0.26, 0.87)**	**0.46 (0.19, 1.0)**	0.83 (0.43, 1.6)	1.0 (0.47, 2.1)	0.73 (0.33, 1.5)
**1.7 (1.1, 2.6)**	**2.0 (1.2, 3.8)**	Stanford V	0.93 (0.41, 2.1)	1.6 (0.73, 4.2)	**2.1 (1.0, 4.4)**	1.5 (0.69, 3.2)
1.8 (0.89, 3.7)	2.2 (0.97, 5.2)	1.1 (0.47, 2.4)	MOPP	1.8 (0.65, 5.4)	**2.2 (1.1, 4.7)**	1.6 (0.88, 2.9)
1.0 (0.43, 2.1)	1.2 (0.61, 2.3)	0.61 (0.24, 1.4)	0.56 (0.18, 1.5)	COPP + ABVD	1.3 (0.42, 3.1)	0.89 (0.30, 2.3)
0.80 (0.43, 1.5)	0.97 (0.47, 2.1)	**0.48 (0.23, 1.0)**	**0.45 (0.21, 0.93)**	0.79 (0.33, 2.4)	MOPP + ABV	0.71 (0.44, 1.2)
1.1 (0.60, 2.1)	1.4 (0.66, 3.1)	0.68 (0.31, 1.5)	0.63 (0.35, 1.1)	1.1 (0.43, 3.3)	1.4 (0.85, 2.3)	MOPP + ABVD
PR						
ABVD	1.1 (0.35, 3.5)	1.6 (0.71, 3.7)	1.3 (0.36, 4.4)	6.9 (0.42, 1.8e+02)	0.83 (0.28, 2.3)	1.0 (0.33, 3.1)
0.94 (0.28, 2.8)	BEACOPP	1.5 (0.36, 6.2)	1.2 (0.21, 6.5)	6.4 (0.55, 1.4e+02)	0.78 (0.15, 3.6)	0.95 (0.18, 4.7)
0.61 (0.27, 1.4)	0.66 (0.16, 2.8)	Stanford V	0.78 (0.18, 3.6)	4.3 (0.23, 1.2e+02)	0.50 (0.13, 2.0)	0.61 (0.16, 2.4)
0.77 (0.23, 2.8)	0.82 (0.15, 4.7)	1.3 (0.28, 5.6)	MOPP	5.4 (0.25, 1.8e+02)	0.64 (0.19, 2.1)	0.78 (0.30, 2.0)
0.15 (0.0054, 2.4)	0.16 (0.0069, 1.8)	0.23 (0.0081, 4.3)	0.19 (0.0054, 3.9)	COPP + ABVD	0.12 (0.0038, 2.2)	0.14 (0.0044, 2.9)
1.22 (0.42, 3.68)	1.34 (0.26, 6.75)	1.84 (0.45, 6.82)	1.61 (0.46, 5.55)	9.43 (0.41, 424.39)	MOPP + ABV	1.23 (0.51, 2.93)
1.0 (0.33, 3.0)	1.1 (0.21, 5.5)	1.6 (0.41, 6.4)	1.3 (0.49, 3.3)	7.1 (0.34, 2.3e+02)	0.82 (0.33, 2.0)	MOPP + ABVD
ORR						
ABVD	1.9 (0.85, 7.0)	0.87 (0.44, 1.7)	0.42 (0.15, 1.3)	5.2 (0.99, 39.0)	1.2 (0.52, 2.9)	0.75 (0.33, 2.0)
0.52 (0.14, 1.2)	BEACOPP	0.45 (0.099, 1.3)	**0.22 (0.041, 0.76)**	2.7 (0.53, 13.0)	0.61 (0.12, 1.9)	0.38 (0.077, 1.3)
1.1 (0.60, 2.3)	2.2 (0.76, 10.0)	Stanford V	0.48 (0.15, 1.8)	**5.8 (1.0, 48.0)**	1.4 (0.49, 4.1)	0.85 (0.30, 2.9)
2.4 (0.79, 6.9)	**4.6 (1.3, 24.0)**	2.1 (0.56, 6.8)	MOPP	**14.0(1.6, 1.1e+02)**	**3.0 (1.0, 7.7)**	1.9 (0.80, 4.0)
**0.19 (0.026, 1.0)**	0.37 (0.077, 1.9)	**0.17 (0.021, 1.0)**	**0.074 (0.0094, 0.62)**	COPP + ABVD	0.21 (0.028, 1.5)	**0.14 (0.019, 0.97)**
0.85 (0.34, 1.9)	1.6 (0.54, 8.7)	0.73 (0.24, 2.0)	**0.34 (0.13, 0.99)**	4.7 (0.65, 36.0)	MOPP + ABV	0.64 (0.32, 1.3)
1.3 (0.51, 3.1)	2.7 (0.79, 13.0)	1.2 (0.35, 3.3)	0.53 (0.25, 1.3)	**7.2 (1.0, 53.0)**	1.6 (0.74, 3.1)	MOPP + ABVD
OS						
ABVD	1.1 (0.61, 1.8)	1.2 (0.77, 1.9)	1.4 (0.87, 2.4)	1.3 (0.53, 2.6)	1.1 (0.65, 1.6)	1.1 (0.66, 1.6)
0.91 (0.55, 1.7)	BEACOPP	1.1 (0.56, 2.3)	1.3 (0.64, 2.8)	1.2 (0.66, 1.8)	0.96 (0.48, 2.0)	0.95 (0.48, 2.0)
0.86 (0.54, 1.3)	0.94 (0.43, 1.8)	Stanford V	1.2 (0.61, 2.3)	1.1 (0.37, 2.4)	0.90 (0.45, 1.6)	0.90 (0.46, 1.6)
0.69 (0.42, 1.2)	0.76 (0.36, 1.6)	0.80 (0.44, 1.6)	MOPP	0.88 (0.33, 2.0)	0.72 (0.42, 1.3)	0.73 (0.46, 1.1)
0.79 (0.39, 1.9)	0.87 (0.54, 1.5)	0.91 (0.42, 2.7)	1.1 (0.51, 3.1)	COPP + ABVD	0.83 (0.37, 2.2)	0.82 (0.36, 2.1)
0.95 (0.61, 1.5)	1.0 (0.50, 2.1)	1.1 (0.62, 2.2)	1.4 (0.80, 2.4)	1.2 (0.46, 2.7)	MOPP + ABV	1.0 (0.67, 1.5)
0.95 (0.63, 1.5)	1.1 (0.51, 2.1)	1.1 (0.62, 2.2)	1.4 (0.90, 2.2)	1.2 (0.47, 2.8)	1.0 (0.66, 1.5)	MOPP + ABVD

Notes: CR, complete response; PR, partial response; ORR, overall remission rate; OS, overall survival; HR, hazard ratio; OR, odds radio; 95%CI, 95%confidence intervals; ABVD, doxorubicin + bleomycin + vinblastine + dacarbazine; BEACOPP, bleomycin + etoposide + doxorubicin + cyclophosphamide + vincristine + procarbazine + prednisone; Stanford V, doxorubicin + vinblastine + mechlorethamine + vincristine + bleomycin + etoposide + prednisone; MOPP, mechlorethamine + vincristine + procarbazine + prednisone; COPP + ABVD, cyclophosphamide + vincristine + procarbazine + prednisone + doxorubicin + bleomycin + vinblastine + dacarbazine; MOPP + ABV(Hybrid), mechlorethamine + vincristine + procarbazine + prednisone + doxorubicin + bleomycin + vinblastine; significant difference is shown in bold and underline formats; the numerical value of each line represents the OR value and CI; OD value >1 indicates that the intervention in the corresponding column is relatively good.

### SUCRA Curves of Chemotherapy Efficacies and Toxicities

The result of SUCRA curve was shown in Table 4, the MOPP + ABV regimen had the highest SUCRA CR value (85.30%), and COPP + ABVD had the highest SUCRA PR (93.47%) and ORR (95.20%) values. The MOPP regimen had the highest SUCRA OS value (74.26%). The ABVD regimen had the highest SUCRA value of anemia (93.81%), thrombocytopenia (90.42%), neutropenia (90.21%) and leucopenia (95.63%), indicating that ABVD has the lowest incidence of anemia, thrombocytopenia, neutropenia and leucopenia. The BEACOPP regimen had the highest SUCRA value of nausea/vomiting (78.03%), indicating that BEACOPP had lowest nausea/vomiting.

**TABLE 4:** SUCRA values of seven chemotherapy regimens under nine outcomes.

Treatments	SUCRA values (%)								
	CR	PR	ORR	OS	Anemia	Thrombocytopenia	Neutropenia	Leucopenia	Nausea/vomiting
A	64.11	43.99	56.00	38.35	**93.81**	**90.42**	**90.21**	**95.63**	47.95
B	81.41	51.56	83.44	52.76	48.6	34.13	64.41	35.73	**78.03**
C	27.49	72.11	43.02	61.59	25.58	79.83	81.97	73.31	61.71
D	23.65	60.51	18.35	**84.26**	43.76	30.92	48.67	NR	62.65
E	63.12	93.47	95.2	68.49	77.42	57.66	NR	45.33	45.08
F	**85.3**	32.95	65.69	47.62	58.46	65.56	30.62	NR	71.58
G	54.91	45.41	38.31	46.91	52.37	41.47	34.12	NR	33.01

Notes: SUCRA, surface under the cumulative ranking curves; NR, not report; CR, complete response; OS, overall survival; PR, partial response; ORR, overall remission rate;A, ABVD (doxorubicin + bleomycin + vinblastine + dacarbazine); B, BEACOPP (bleomycin + etoposide + doxorubicin + cyclophosphamide + vincristine + procarbazine + prednisone); C, StanfordV (doxorubicin + vinblastine + mechlorethamine + vincristine + bleomycin + etoposide + prednisone); D, MOPP (mechlorethamine + vincristine + procarbazine + prednisone); E, COPP + ABVD (cyclophosphamide + vincristine + procarbazine + prednisone + doxorubicin + bleomycin + vinblastine + dacarbazine); F, MOPP + ABV (Hybrid) (mechlorethamine + vincristine + procarbazine + prednisone + doxorubicin + bleomycin + vinblastine); G, MOPP + ABVD(Alternating); The best intervention is shown in bold and underline formats.

### SUCRA Value Cluster Analyses

**FIGURE 6:**
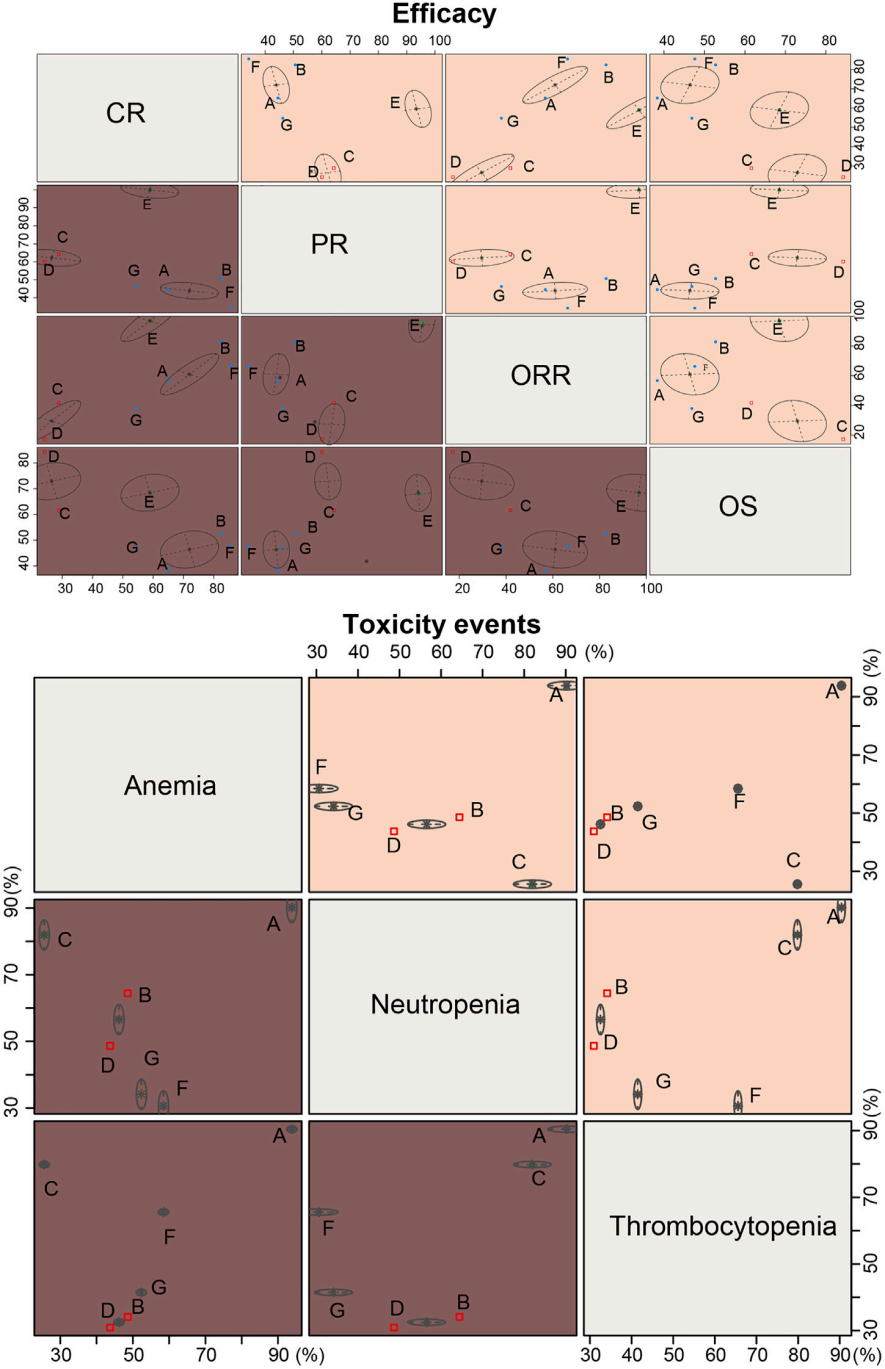
Cluster ranking plots based on SUCRA efficacy and toxicity values of seven chemotherapy regimens for HL. A = ABVD (doxorubicin + bleomycin + vinblastine + dacarbazine); B = BEACOPP (bleomycin + etoposide + doxorubicin + cyclophosphamide + vincristine + procarbazine + prednisone); C = Stanford V (doxorubicin + vinblastine + mechlorethamine + vincristine + bleomycin + etoposide + prednisone); D = MOPP (mechlorethamine + vincristine + procarbazine + prednisone);E = COPP + ABVD (cyclophosphamide + vincristine + procarbazine + prednisone + doxorubicin + bleomycin + vinblastine + dacarbazine); F = MOPP + ABV (Hybrid) (mechlorethamine + vincristine + procarbazine + prednisone + doxorubicin + bleomycin + vinblastine); G = MOPP + ABVD (Alternating).

The results of SUCRA value cluster analyses are shown in Figure 6. In chemotherapy efficacy (CR, PR, ORR, and OS), the COPP + ABVD regimen was most effective among the seven regimens. In toxicity (anemia, neutropenia and thrombocytopenia), BEACOPP, MOPP, and MOPP + ABVD had higher toxicities than the other regimens, and ABVD was less toxic.

## Discussion

Our NMA compared the outcomes of seven chemotherapy regimens across 14 RCTs involving 5,964 HL patients. Our results demonstrated that, compared with other regimens, COPP + ABVD produced the best outcomes, with high SUCRA PR and ORR values. However, the ABVD regimen exhibited the lowest toxicity rates. ABVD is the most widely used regimen for advanced HL patients (Corazzelli et al., 2011). After the MOPP regimen, ABVD was superior to or less toxic than non-cross-resistant alternating regimens (MOPP + ABVD and MOPP + ABV), multidrug regimens (MOPP) and chemoradiotherapy (Stanford V) (Ansell, 2016; Carde et al., 2016). Thus, ABVD is still the standard HL treatment, with a good balance of efficacy and toxicity (Chisesi et al., 2011).

Our results indicated that the MOPP + ABV regimen had the highest SUCRA CR value. At the time the current trial was planned, randomized trials comparing MOPP + ABV (hybrid) treatment with both MOPP to ABVD (sequential) and MOPP + ABVD (alternating) therapy had recently begun. Some trials demonstrated that same effectiveness between the MOPP + ABV (hybrid) and MOPP_+ ABVD (alternating) which are more effective than sequential MOPP to ABVD treatment (Santoro et al., 1987; Connors et al., 1997). Additionally, MOPP + ABV have relationship with greater incidences of acute toxicity, MDS, and leukemia (Duggan et al., 2003).

Besides, our study also showed that the BEACOPP regimen had the lowest incidence of nausea/vomiting in the seven chemotherapy regimens. As the larger the SUCRA value was, the better the rank of the intervention was, BEACOPP was the best regimen for the outcome-nausea/vomiting. Nausea/vomiting were bad outcomes, so the lower incidence they had, the better the regimen was. As an alternative to escalated BEACOPP therapy, patients who have not experienced CR with more standard ABVD chemotherapy can receive an autologous stem cell transplant (Majhail et al., 2006). Our NMA indicated that the BEACOPP, MOPP, and MOPP + ABVD regimens exhibited higher toxicities than the other regimens. Toxicities related to BEACOPP included hematological toxicity and infection, and longer-term risks which including infertility, stem cell injury, and leukemia (Ansell, 2016). The escalated BEACOPP regimen has been proved that it can lead to more haematological toxicities WHO grade III or IV (Skoetz et al., 2017). Six cycles of escalated BEACOPP regimen significantly improves OS compared with ABVD and other regimens (Skoetz et al., 2013). The point of our study is the efficacy between different drugs rather than different doses of the same drug. Besides, the outcome of our article is various, focusing not only on the efficacy but also on the safety.

Furthermore, we used a Bayesian network model to assess direct and indirect evidence inconsistency *via* the node-splitting method. This method allowed us to eliminate potential errors of the NMA and further compare the seven interventions (Yan et al., 2015). In comparison with the Skoetz N et al. Lancet 2013 study, our study has made some improvements. First, Skoetz N et al. Lancet 2013 study focused on overall survival but our study involved overall survival, complete response, partial response, overall remission rate, anemia, thrombocytopenia, neutropenia, leukopenia and nausea/vomiting. Second, in Skoetz N et al. Lancet 2013 study, compared with overall survival for ABVD, overall survival for each regimen was not differed as presented in their Figure 3. But in our study, we supplemented SUCRA values. From the SUCRA values, we could infer that people receive MOPP regimen have longer term overall survival, followed by COPP + ABVD. Third, we adopted cluster analysis of SUCRA values to find the best regimen from all outcomes and we illustrated that COPP + ABVD was considered as the best regimen based on comprehensive analysis. Last, in our study, SUCRA values were concluded from comparisons among all regimens and the largest SUCRA values indicated the best regimen. While SUCRA values were not mentioned in Skoetz N et al. Lancet 2013 study, so the best regimen could not be suggested. Nevertheless, our study had several limitations. First, the number of included studies was relatively small, and there was no cross-research comparison. Second, patient cohort sizes differed between the seven chemotherapy regimens, which may have biased our NMA results and reduced the accuracy of our findings (Jansen, 2012; Saramago et al., 2012). Third, we could not statistically analyze PFS indicators, since only 4/14 included studies provided PFS indicator information. Forth, nausea/vomiting were excluded from the toxicity analysis because comparisons among different regimens about this outcome were not statistically different. Further toxicity analysis about nausea/vomiting need to be investigated in the future. Finally, treatment-related toxicity, especially pulmonary and cardiac toxicity and infection (Diehl et al., 2003), is more common in older patients. Only 4/14 studies mentioned pulmonary toxicity due to bleomycin treatment, and only 3/14 discussed treatment related mortality rates, which limited our ability to assess treatment-related toxicity.

In summary, we found that the COPP + ABVD regimen had the best efficacy in HL patients, and ABVD with the lowest toxicity. At present, PET-CT is mostly used as the main way to evaluate CR, while our study used CT as the main way to evaluate CR about the included literature, which might affect our final result. Additionally, statistical analysis of PFS indicators cannot be carried out since only 4 of the 14 literatures had PFS indicators. Furthermore, treatment-related toxicity played a vital role among old patients, especially the risk of pulmonary cardiac, toxicity and infection, while only 4 enrolled studies referred to bleomycin due to pulmonary toxicity and others did not mentioned for statistical analysis; only three enrolled studies referred to treatment related mortality rate and others did not mention for statistical analysis. While our study included a large total number of patients, and our results were in agreement with other groups’ findings, our conclusions must be confirmed by additional studies with large sample sizes and broader multivariate analyses.

## Supplementary Material

Supplementary Figure S1 Flow chart showing literature search and study selection. Fifteen randomized controlled trials met the inclusion criteria and were included in this meta-analysis.
