# Supplementary material for: Efficacies and Toxicities of Seven Chemotherapy Regimens for Advanced Hodgkin Lymphoma
Source: Front Pharmacol. 2021 Nov 16;12:694545. doi: 10.3389/fphar.2021.694545 (PMC8635017; doi:10.3389/fphar.2021.694545)
Supplement: Supplementary file 1 [file Table1.docx]

**Supplementary table 1. The baseline characteristics for included studies.**

| **First author** | **Country** | **Interventions** | | | **Age (years)** |  |  | **Sample size** |  | |  | CR Assessment |
| --- | --- | --- | --- | --- | --- | --- | --- | --- | --- | --- | --- | --- |
|  |  | T1 | T2 | T3 | T1 | T2 | T3 | T1 | T2 | T3 | |  |
| Carde P(2016) | France | ABVD | BEACOPP |  | 34.9 (16.1-67.4) | 35.3 (16.8-60.9) |  | 275 | 274 |  | | CT |
| Mounier N(2014) | France | ABVD | BEACOPP |  | 28(16–60) | 28(16–68) |  | 80 | 70 |  | | CT |
| Gordon LI(2013) | America | ABVD | Stanford V |  | 33(18–83) | 33(16–83) |  | 395 | 399 |  | | CT |
| Viviani S(2011) | Italy | ABVD | BEACOPP |  | NR | NR |  | 168 | 163 |  | | CT/PET |
| Hoskin PJ(2009) | America | ABVD | Stanford V |  | 35(18-60) | 34(18-67) |  | 261 | 259 |  | | CT |
| Federico M(2009) | Italy | ABVD | BEACOPP |  | 32 | 29 |  | 99 | 98 |  | | CT |
| Gobbi PG(2005) | Italy | ABVD | Stanford V |  | 31(16-68) | 34(17-69) |  | 122 | 107 |  | | CT |
| Ballova V(2005) | Germany | BEACOPP | COPP + ABVD |  | 69(66-75) | 70(66-75) |  | 42 | 26 |  | | CT |
| Duggan DB(2003) | Canada | ABVD | MOPP/ABV |  | NR | NR |  | 433 | 419 |  | | CT |
| Diehl V(2003) | Germany | BEACOPP | COPP + ABVD |  | 32.7 | 32.1 |  | 469 | 260 |  | | CT |
| Glick JH(1998) | America | MOPP/ABV | MOPP + ABVD |  | 30.7 | 30.8 |  | 347 | 344 |  | | CT |
| Connors JM(1997) | Canada | MOPP/ABV | MOPP + ABVD |  | 30.2 | 30.6 |  | 153 | 148 |  | | CT/biopsy |
| Somers R(1994) | Netherlands | MOPP | MOPP + ABVD |  | 33 ± 12 | 35 ± 13 |  | 96 | 96 |  | | CT |
| Canellos GP(1992) | America | ABVD | MOPP | MOPP + ABVD | 35(16-71) | 34(16-72) | 32(16-72) | 115 | 123 | 123 | | X-ray |

Notes: T = treatment; NR = not report; CT = computed tomography; PET = positron emission tomography; ABVD = doxorubicin + bleomycin + vinblastine + dacarbazine; BEACOPP = bleomycin + etoposide + doxorubicin + cyclophosphamide + vincristine + procarbazine + prednisone; StanfordV = doxorubicin + vinblastine + mechlorethamine + vincristine + bleomycin + etoposide + prednisone; MOPP = mechlorethamine + vincristine + procarbazine + prednisone; COPP + ABVD = cyclophosphamide + vincristine + procarbazine + prednisone + doxorubicin + bleomycin + vinblastine + dacarbazine; MOPP + ABV(Hybrid) = mechlorethamine + vincristine + procarbazine + prednisone + doxorubicin + bleomycin + vinblastine.
