# Supplementary figures and images for: Efficacies and Toxicities of Seven Chemotherapy Regimens for Advanced Hodgkin Lymphoma
Source: Front Pharmacol. 2021 Nov 16;12:694545. doi: 10.3389/fphar.2021.694545 (PMC8635017; doi:10.3389/fphar.2021.694545)

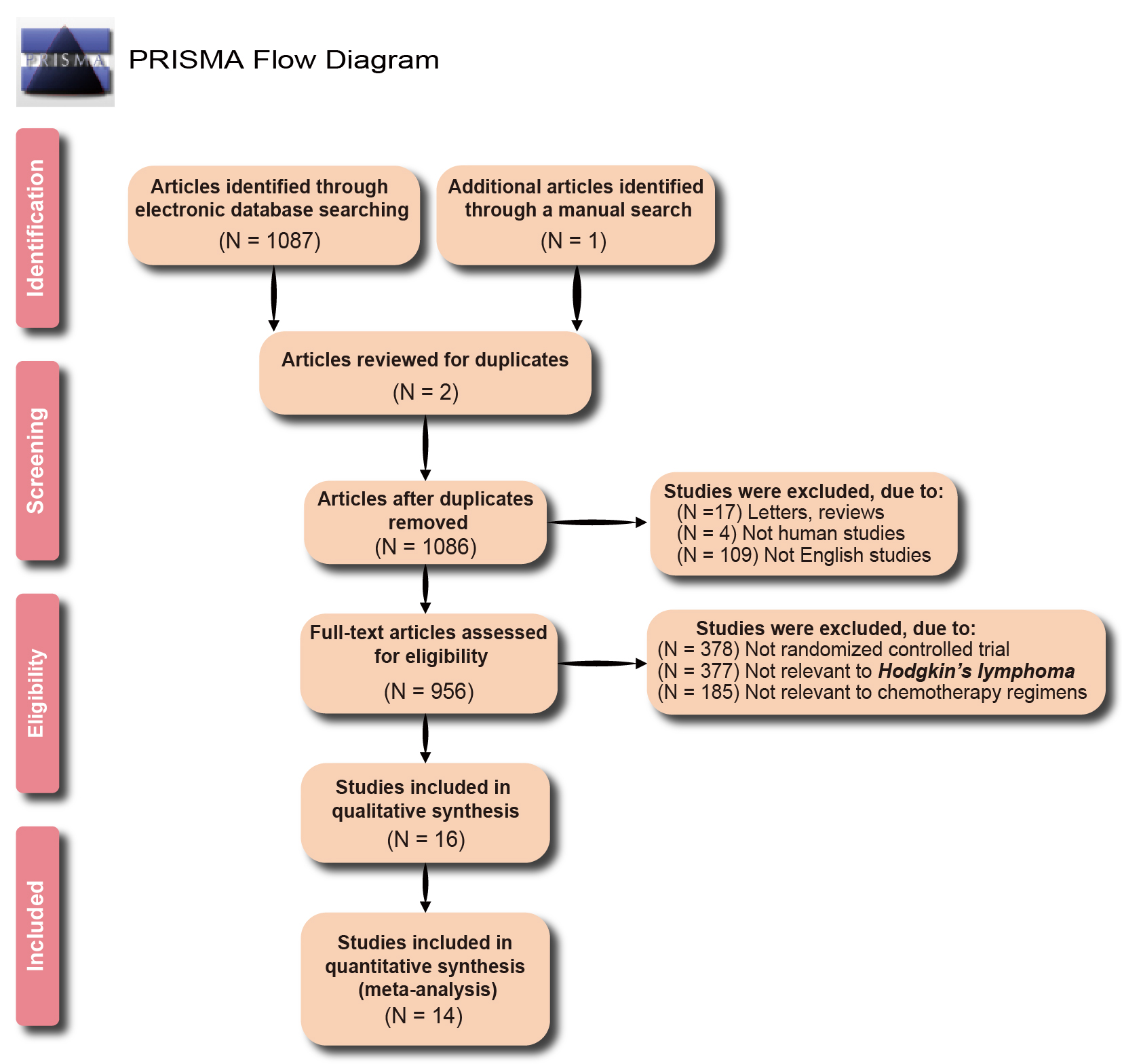

Supplement: Supplementary file 2 [file Image1.jpeg]
