# Supplementary material for: Efficacies and Toxicities of Seven Chemotherapy Regimens for Advanced Hodgkin Lymphoma
Source: Front Pharmacol. 2021 Nov 16;12:694545. doi: 10.3389/fphar.2021.694545 (PMC8635017; doi:10.3389/fphar.2021.694545)
Supplement: Supplementary file 3 [file Table2.docx]

| **Supplementary table 2. OR (95%CI) of seven chemotherapy regimens of five endpoints.** | | | |  |  |  |
| --- | --- | --- | --- | --- | --- | --- |
| **OR (95%CI)** | | | | | | |
| **Anemia** |  |  |  |  |  |  |
| ABVD | **7.13 (1.12, 54.77)** | **31.87 (1.44, 1548.37)** | 8.85 (0.67, 138.76) | 2.17 (0.16, 36.90) | 5.66 (0.15, 262.43) | 6.74 (0.46, 125.78) |
| **0.14 (0.02, 0.90)** | BEACOPP | 4.50 (0.09, 337.16) | 1.25 (0.04, 36.07) | 0.31 (0.04, 2.11) | 0.80 (0.01, 54.35) | 0.93 (0.03, 27.01) |
| **0.03 (0.00, 0.69)** | 0.22 (0.00, 11.32) | Stanford V | 0.28 (0.00, 17.71) | 0.07 (0.00, 4.50) | 0.18 (0.00, 23.70) | 0.21 (0.00, 12.32) |
| 0.11 (0.01, 1.50) | 0.80 (0.03, 22.35) | 3.56 (0.06, 370.01) | MOPP | 0.24 (0.01, 11.96) | 0.63 (0.01, 24.45) | 0.74 (0.05, 9.72) |
| 0.46 (0.03, 6.34) | 3.27 (0.47, 23.24) | 14.76 (0.22, 1679.86) | 4.15 (0.08, 177.06) | COPP+ABVD | 2.65 (0.02, 228.13) | 3.07 (0.06, 137.71) |
| 0.18 (0.00, 6.76) | 1.25 (0.02, 91.39) | 5.54 (0.04, 1288.45) | 1.58 (0.04, 67.87) | 0.38 (0.00, 41.45) | MOPP+ABV | 1.19 (0.09, 15.83) |
| 0.15 (0.01, 2.19) | 1.07 (0.04, 31.94) | 4.78 (0.08, 563.19) | 1.34 (0.10, 19.32) | 0.33 (0.01, 16.26) | 0.84 (0.06, 10.82) | MOPP+ABVD |
| **Thrombocytopenia** |  |  |  |  |  |  |
| ABVD | **17.54 (3.49, 136.87)** | 1.08 (0.02, 94.60) | **21.38 (1.53, 333.04)** | 7.28 (0.59, 120.78) | 4.97 (0.12, 226.87) | 13.70 (0.91, 214.19) |
| **0.06 (0.01, 0.29)** | BEACOPP | 0.06 (0.00, 6.78) | 1.21 (0.04, 25.84) | 0.41 (0.06, 2.66) | 0.28 (0.00, 16.24) | 0.77 (0.02, 17.69) |
| 0.93 (0.01, 61.14) | 17.60 (0.15, 1521.64) | Stanford V | 19.92 (0.11, 2772.17) | 6.97 (0.04, 955.49) | 4.63 (0.02, 1041.98) | 12.97 (0.08, 1643.53) |
| **0.05 (0.00, 0.65)** | 0.82 (0.04, 25.39) | 0.05 (0.00, 8.90) | MOPP | 0.34 (0.01, 13.75) | 0.23 (0.01, 9.71) | 0.65 (0.04, 8.25) |
| 0.14 (0.01, 1.70) | 2.46 (0.38, 17.20) | 0.14 (0.00, 25.27) | 2.94 (0.07, 131.29) | COPP+ABVD | 0.68 (0.01, 70.92) | 1.90 (0.04, 78.85) |
| 0.20 (0.00, 8.08) | 3.56 (0.06, 253.60) | 0.22 (0.00, 63.14) | 4.31 (0.10, 185.69) | 1.46 (0.01, 160.12) | MOPP+ABV | 2.76 (0.19, 36.24) |
| 0.07 (0.00, 1.09) | 1.29 (0.06, 40.36) | 0.08 (0.00, 12.77) | 1.54 (0.12, 23.56) | 0.53 (0.01, 26.67) | 0.36 (0.03, 5.35) | MOPP+ABVD |
| **Neutropenia** |  |  |  |  |  |  |
| ABVD | 3.08 (0.34, 26.79) | 1.22 (0.06, 24.21) | 8.31 (0.43, 166.63) | 21.38 (0.28, 1123.31) | 16.75 (0.77, 307.94) |  |
| 0.32 (0.04, 2.90) | BEACOPP | 0.40 (0.01, 15.93) | 2.77 (0.06, 112.83) | 7.07 (0.05, 636.12) | 5.57 (0.12, 193.18) |  |
| 0.82 (0.04, 17.00) | 2.48 (0.06, 107.36) | Stanford V | 6.70 (0.09, 493.37) | 17.45 (0.09, 2534.17) | 13.61 (0.18, 914.32) |  |
| 0.12 (0.01, 2.32) | 0.36 (0.01, 15.57) | 0.15 (0.00, 10.61) | MOPP | 2.55 (0.04, 148.76) | 2.00 (0.10, 38.17) |  |
| 0.05 (0.00, 3.60) | 0.14 (0.00, 19.39) | 0.06 (0.00, 11.17) | 0.39 (0.01, 27.44) | MOPP+ABV | 0.78 (0.04, 16.71) |  |
| 0.06 (0.00, 1.30) | 0.18 (0.01, 8.16) | 0.07 (0.00, 5.42) | 0.50 (0.03, 10.30) | 1.28 (0.06, 22.98) | MOPP+ABVD |  |
| **Leucopenia** |  |  |  |  |  |  |
| ABVD | **23.0 (2.7, 1.9e+02)** | 2.9 (0.29, 35.0) | 15.0 (0.67, 3.8e+02) |  |  |  |
| **0.044 (0.0051, 0.36)** | BEACOPP | 0.13 (0.0048, 2.9) | 0.66 (0.053, 8.2) |  |  |  |
| 0.34 (0.028, 3.4) | 7.6 (0.35, 2.1e+02) | Stanford V | 5.1 (0.097, 3.3e+02) |  |  |  |
| 0.066 (0.0026, 1.5) | 1.5 (0.12, 19.0) | 0.20 (0.0031, 10.0) | COPP+ABVD |  |  |  |
| **Nausea/vomiting** |  |  |  |  |  |  |
| ABVD | 0.56 (0.17, 2.0) | 0.76 (0.16, 3.0) | 0.79 (0.28, 2.2) | 1.1 (0.24, 4.9) | 0.61 (0.15, 2.6) | 1.3 (0.46, 3.5) |
| 1.8 (0.50, 5.9) | BEACOPP | 1.2 (0.17, 8.2) | 1.4 (0.27, 7.0) | 1.9 (0.72, 4.8) | 1.1 (0.16, 7.0) | 2.3 (0.46, 10.0) |
| 1.3 (0.34, 6.4) | 0.80 (0.12, 5.8) | Stanford V | 1.0 (0.19, 7.1) | 1.5 (0.20, 13.0) | 0.83 (0.12, 6.3) | 1.8 (0.32, 10.0) |
| 1.3 (0.46, 3.6) | 0.73 (0.14, 3.6) | 0.96 (0.14, 5.2) | MOPP | 1.3 (0.22, 9.4) | 0.78 (0.19, 3.3) | 1.7 (0.61, 4.7) |
| 0.93 (0.20, 4.2) | 0.54 (0.21, 1.4) | 0.69 (0.077, 4.9) | 0.75 (0.11, 4.5) | COPP+ABVD | 0.57 (0.072, 4.7) | 1.3 (0.19, 7.2) |
| 1.6 (0.39, 6.5) | 0.94 (0.14, 6.2) | 1.2 (0.16, 8.4) | 1.3 (0.30, 5.2) | 1.7 (0.21, 14.0) | MOPP+ABV | 2.2 (0.75, 5.8) |
| 0.75 (0.29, 2.2) | 0.43 (0.096, 2.2) | 0.56 (0.096, 3.1) | 0.59 (0.21, 1.6) | 0.80 (0.14, 5.2) | 0.46 (0.17, 1.3) | MOPP+ABVD |
| Notes: OR=odds radio;95%CI=95%confidence intervals;ABVD=doxorubicin+bleomycin+vinblastine+dacarbazine;BEACOPP=bleomycin+etoposide+doxorubicin+cyclophosphamide +vincristine+ procarbazine+prednisone;Stanford V=doxorubicin+vinblastine+mechlorethamine+vincristine+bleomycin+etoposide+prednisone; MOPP=mechlorethamine+vincristine+procarbazine+prednisone;COPP+ABVD=cyclophosphamide+vincristine+procarbazine+prednisone+doxorubicin+bleomycin+vinblastine+dacarbazine;MOPP+ABV(Hybrid)=mechlorethamine+vincristine+procarbazine+prednisone+doxorubicin+bleomycin+vinblastine; significant difference is shown in bold and underline formats; the numerical value of each line represents the OR value and CI; OD value > 1 indicates that the intervention in the corresponding column is relatively poor. | | | | | | |
|  |  |  |  |  |  |  |
|  |  |  |  |  |  |  |
|  |  |  |  |  |  |  |
|  |  |  |  |  |  |  |
