# Supplementary material for: Efficacies and Toxicities of Seven Chemotherapy Regimens for Advanced Hodgkin Lymphoma
Source: Front Pharmacol. 2021 Nov 16;12:694545. doi: 10.3389/fphar.2021.694545 (PMC8635017; doi:10.3389/fphar.2021.694545)
Supplement: Supplementary file 4 [file DataSheet1.docx]

**Search Strategy**

1."Hodgkin Disease"[Mesh]OR Hodgkin's Granuloma[tiab] OR Hodgkins Granuloma[tiab] OR Malignant Lymphogranuloma[tiab] OR Hodgkin Lymphoma[tiab] OR Hodgkin's Disease[tiab] OR Hodgkin's Lymphoma[tiab] OR Hodgkins Lymphoma[tiab] OR Hodgkins Disease[tiab] OR Hodgkin Granuloma[tiab] OR Malignant Granuloma[tiab]

2.chemotherapy drugs/Antineoplastic Agents

#1 "lymphoma"[mh] OR Lymphomas[tiab] OR Sarcoma, Germinoblastic[tiab] OR Germinoblastic Sarcomas[tiab] OR Reticulolymphosarcoma[tiab] OR Reticulolymphosarcomas[tiab] OR Germinoblastoma[tiab] OR Germinoblastomas[tiab] OR Lymphoma, Malignant[tiab] OR Lymphomas, Malignant[tiab] OR Malignant Lymphoma[tiab]

#2 "randomized controlled trial"[pt] OR "controlled clinical trial"[pt] OR "randomized controlled trials as topic"[mh] OR "clinical trials as topic"[mh] OR "controlled clinical trials as topic"[mh] OR placebos[mh] OR "random allocation"[mh] OR “double-blind method"[mh] OR randomized[tiab] OR placebo[tiab] OR randomization[tiab] OR randomly allocated[tiab] OR ((double[tw] OR treble[tw] OR triple[tw]) AND (mask* [tw] OR blind* [tw]))

#4 "Ifosfamide"[mh] OR Iphosfamide[tiab] OR "Carboplatin"[mh] OR "Etoposide"[mh] OR "dexamethasone" [mh] OR "Cisplatin" [mh] OR "Methotrexate"[mh] OR "mitoguazone" [Supplementary Concept] OR "methylprednisolone"[Supplementary Concept] OR "Cytarabine"[mh] OR "DHAP protocol" [Supplementary Concept] OR "ICE protocol"[Supplementary Concept] OR "MIME protocol"[Supplementary Concept] OR "ESAP protocol"[Supplementary Concept] OR "ABVD protocol"[Supplementary Concept] OR "MOPP protocol"[Supplementary Concept] OR "Chlorambucil"[mh] OR "cyclophosphamide"[mh] OR "Mitoxantrone" [mh] OR "CHVP protocol"[Supplementary Concept] OR "Dacarbazine" [mh] OR "Doxorubicin"[mh] OR "Vinblastine" [mh] OR Bleomycin{tiab] OR "Vincristine"[mh] OR "Procarbazine"[mh] OR "Prednisone"[mh] OR "Mechlorethamine"[mh] OR "Fludarabine"[mh] OR "Mitoxantrone"[mh] OR "Pentostatin" [mh] OR "Thalidomide"[mh]OR chemotherapy[tiab]OR Stanford V[tiab]OR MOPP[tiab]OR COPP[tiab]OR BEACOPP[tiab]OR"Etoposide"[Mesh]

#5 “meta-analysis"[pt] OR “meta-analysis as topic"[mh] OR meta-analysis[tiab] OR network meta-analysis[tiab] OR mixed treatment comparison*[tiab] OR multiple treatment comparison*[tiab] OR multiple treatment meta-analysis[tiab]
